# Levetiracetam Affects Differentially Presynaptic Proteins in Rat Cerebral Cortex

**DOI:** 10.3389/fncel.2017.00389

**Published:** 2017-12-11

**Authors:** Daniele Marcotulli, Giorgia Fattorini, Luca Bragina, Jessica Perugini, Fiorenzo Conti

**Affiliations:** ^1^Department of Experimental and Clinical Medicine, Università Politecnica delle Marche, Ancona, Italy; ^2^Center for Neurobiology of Aging, INRCA IRCCS, Ancona, Italy

**Keywords:** levetiracetam, vesicular transport proteins, SV2A, presynaptic proteins, interactome, LRRK2

## Abstract

Presynaptic proteins are potential therapeutic targets for epilepsy and other neurological diseases. We tested the hypothesis that chronic treatment with the SV2A ligand levetiracetam affects the expression of other presynaptic proteins. Results showed that in rat neocortex no significant difference was detected in SV2A protein levels in levetiracetam treated animals compared to controls, whereas levetiracetam post-transcriptionally decreased several vesicular proteins and increased LRRK2, without any change in mRNA levels. Analysis of SV2A interactome indicates that the presynaptic proteins regulation induced by levetiracetam reported here is mediated by this interactome, and suggests that LRRK2 plays a role in forging the pattern of effects.

## Introduction

Levetiracetam (LEV), a broad-spectrum anti-epileptic drug approved by FDA in 1999, is widely prescribed for the treatment of partial and generalized epilepsy (Kaminski et al., 2012), and is attracting growing interest in the therapy of other diseases, including dyskinesia, neuropathic pain, and Alzheimer disease (Crepeau and Treiman, 2010; Sanchez et al., 2012). Lynch et al. (2004) discovered that the synaptic vesicle protein SV2A is the receptor for LEV, a finding confirmed later in SVA2A knockout mice (Garcia-Perez et al., 2015); synaptic activity and concomitant vesicular release allow LEV to enter recycling vesicles to reach SV2A and modulate transmitter release, with marked effects on rapidly discharging neurons (Meehan et al., 2011).

SV2 is a component of all vertebrate synaptic vesicles (SVs) (Lynch et al., 2004; Chang and Sudhof, 2009), where it plays a crucial role in the trafficking of synaptotagmin (SYT) 1, thereby regulating calcium-induced vesicle fusion (Xu et al., 2007). Interestingly, SV2 and SYT1 levels correlate with those of synaptogyrins (SGYRs) (Yao et al., 2010), suggesting that other SV proteins may be influenced by SV2, in agreement with the observation that SV2 proteins function as cargo in co-traffiking of SVs proteins (Yao et al., 2010).

The aim of present study was therefore to verify the hypothesis that chronic LEV treatment induces changes in the expression of SV proteins other than SV2A, in line with the emerging notion that presynaptic proteins are potential therapeutic targets for epilepsy and other neurological diseases (Li and Kavalali, 2017).

## Materials and Methods

### Animals and Treatment

Adult male Sprague-Dawley albino rats (170–200 g; Envigo RMS Srl, Udine, Italy) were used. Their care and handling was approved by the local ethical committee for animal research. All experimental procedures involving animals and their care were carried out in accordance with National laws and policies (D.L. n. 26, March 14, 2014) and with the guidelines established by the European Community Council Directive (2010/63/UE) and were approved by the local authority veterinary services. Animals were kept under a dark-light cycle of 12 h and permitted food and water *ad libitum*.

Rats were randomly divided into two groups. Animals of the first group were administered daily intraperitoneal (i.p.) injections of levetiracetam (54 mg/kg; Keppra, UCB Pharma, Braine-l’Alleud, Belgium; LEV) dissolved in physiological saline at a concentration of 10 mg/ml; those belonging to the second group received the vehicle (physiological saline; 5.4 ml/kg) i.p. (Ueda et al., 2007). All animals received i.p. injections each morning between 09:00 and 11:00; they were sacrificed on the 14th day, 2 h after having received the last i.p. injection.

### Antibodies

Source, concentrations, and data on the characterization of primary and secondary antibodies used in this study are listed in **Tables 1A,B**.

**Table 1A:** Primary antibodies.

Antibodies	Host^∗^	Dilution^∗^	Source	Characterization	RRID
14-3-3β	Rb	1:1000 (WB)	Santa Cruz Biotechnology/sc-628	Wiltfang et al., 1999	AB_630818
14-3-3𝜀	Ms	1:1000 (WB)	Santa Cruz Biotechnology/sc-23957 (8C3)	Raphael et al., 2012	AB_626619
LRRK2	Rb	1:1000 (WB)	Abcam/ab133474	Davies et al., 2013	AB_2713963
Munc18-1	Rb	1:1000 (WB)	Synaptic System/116002	Cijsouw et al., 2014	AB_887736
Rab3a	M	1:1000 (WB)	Synaptic System/107111 (42.2)	Matteoli et al., 1991	AB_887770
Rab3c	Rb	1:1000 (WB)	Synaptic System/107203	Cai et al., 2008	AB_887771
SGYR1	Rb	1:1000 (WB)	Synaptic System/103002	Baumert et al., 1990; Stenius et al., 1995; Von Kriegstein et al., 1999	AB_887818
SGYR3	Rb	1.1000 (WB)	R Janz (Texas University, Houston, United States)	Belizaire et al., 2004	AB_2619752
SNAP25	M	1:3000 (WB)	Serotec/MCA1308 (SP12)	Honer et al., 1997	AB_322417
STX1A	M	1:1000 (WB)	Synaptic System/110111 (78.3)	Varoqueaux et al., 2002	AB_887848
STX1B	Rb	1:1000 (WB)	Synaptic System/110403	Low et al., 2002	AB_887900
SV2A	Rb	1:1000 (WB)	Synaptic System/119002	Janz and Sudhof, 1999	AB_887802
SV2B	Rb	1:1000 (WB)	Synaptic System/119102	Janz and Sudhof, 1999	AB_887803
SYNI	M	1:500 (WB)	F Benfenati (University of Genoa, I) (10.22)	Vaccaro et al., 1997	NR
SYNII	M	1:500 (WB)	F Benfenati (University of Genoa, I) (19.21)	Vaccaro et al., 1997	NR
SYPI	M	1.2000 (WB)	Synaptic System/101011 (7.2)	Jahn et al., 1985	AB_887824
SYT1	M	1:500 (WB)	Synaptic System/105011 (41.1)	Brose et al., 1992; Von Kriegstein et al., 1999	AB_887832
SYT2	Rb	1:1000 (WB)	Synaptic System/105123	Johnson et al., 2010	AB_2199465
SYT9	Rb	1:1000 (WB)	Synaptic System/105053	Dean et al., 2012	AB_2199639
VAMP1	Rb	1:1000 (WB)	Synaptic System/104002	Trimble et al., 1988	AB_887807
VAMP2	M	1:1000 (WB)	Synaptic System/104211 (69.1)	Edelmann et al., 1995	AB_887811
VGAT	Rb	1:500 (IF) 1:1000 (WB)	Synaptic System/131003	Takamori et al., 2000	AB_887869
VGLUT1	GP	1:800 (IF) 1:1000 (WB)	Millipore (Chemicon)/AB5905	Melone et al., 2005	AB_2301751
VGLUT2	GP	1:800 (IF) 1:1000 (WB)	Millipore (Chemicon)/AB2251	Cubelos et al., 2005; Liu et al., 2005	AB_1587626

**Table 1B:** Secondary antibodies.

Conjugated to	React^∗^	Dilution	Source	RRID
Alexa Fluor^®^ 488	GP	1:250	Jackson ImmunoResearch, West Grove, PA/706-546-148	AB_2340473
Cy^TM^3	Rb	1:250	Jackson ImmunoResearch, West Grove, PA/711-166-152	AB_2313568
Peroxidase	GP	1:4000	Jackson ImmunoResearch, West Grove, PA/706-036-148	AB_2340448
Peroxidase	M	1:4000	Jackson ImmunoResearch, West Grove, PA/715-036-151	AB_2340774
Peroxidase	R	1:4000	Jackson ImmunoResearch, West Grove, PA/711-036-152	AB_2340590

*^∗^GP, guinea pig; M, mouse; R, rabbit; IF, immunofluorescence; WB, western blotting; NR, not registered.*

### Western Blotting

**FIGURE 1:**
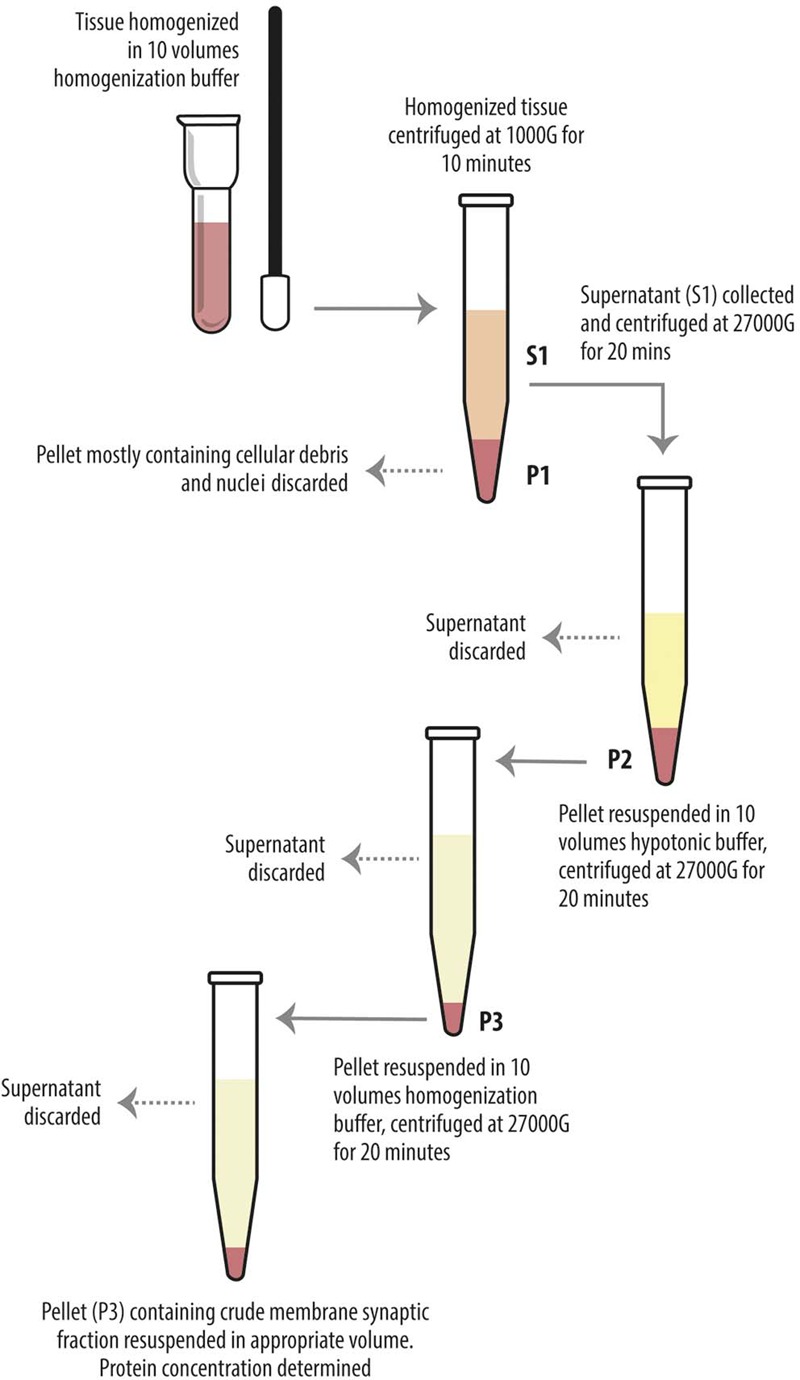
Diagram showing preparatory steps for western blotting samples (S1 and P3), according to Danbolt et al. (1990). The tissue was homogenized in about 10 volumes of homogenization buffer and centrifuged (10 min, 1000 *g*, 4°C). The pellets (P1) were discarded. The combined supernatants (S1) were centrifuged again (20 min, 27000 *g*, 4°C), and the pellets (P2) were suspended in about 10 volumes of hypotonic buffer and centrifuged (20 min, 27000 *g*, 4°C). The pellets (P3) were resuspended in 10 volumes of homogenization buffer in order to eliminate the hypotonic buffer and centrifuged (20 min, 27000 *g*, 4°C). The final pellets (P3) were resuspended in appropiate volume.

Levetiracetam-treated and control rats were anesthetized with chloral hydrate (300 mg/kg i.p.) and decapitated, and cerebral neocortex and hippocampus were quickly separated. Homogenization and crude synaptic plasma membrane preparation were carried out as described (Danbolt et al., 1990). Western blot experiments were carried out on supernatant of the first 1000 *g* centrifuge (S1), containing whole tissue protein content except crude nuclear fraction, blood and other debris (Danbolt et al., 1990; Xu et al., 2013) and on crude membrane synaptic fractions (P3) (Danbolt et al., 1990) (**Figure 1**). Bio-Rad Protein Assay (Bio-Rad Laboratories GmbH, Munchen, Germany) and a Beckman DU 530 spectrophotometer (Beckman Coulter, Fullerton, CA, United States) were used to determine the total amount of protein in each homogenate (3–4 measurements per homogenate). A standard curve with 2, 4, 6, 8, and 10 mg of bovine serum albumin (A4503, Sigma Chemicals, St. Louis, MO, United States) was drawn for each dosing run. As housekeeping proteins (such as α-actin and β-tubulin) are sensitive to experimental treatments (particularly to pharmacologic treatments) and to diverse physiological conditions, and have therefore some limitations as internal standards (Ferguson et al., 2005), 3–6 measurements were made for each brain region of each animal. To minimize procedural variables, homogenates from treated and control animals were loaded onto the same gel (Bragina et al., 2006). For quantitative analysis, we drew standard curves of increasing concentration of total protein from controls to define a linear range for immunoblot densitometric analysis (Bragina et al., 2006); for optimal resolution, western blotting studies were performed in crude synaptic membranes with 7 μg of total protein for each antigen, except for VGLUT2 studies in hippocampal samples and for LRRK2 in P3 of both hippocampus and neocortex, where 15 μg of total protein was used because of the poor antigen expression. Aliquots of crude membrane fraction (P3) or first centrifuge supernatant (S1) from treated and control animals were subjected to SDS-PAGE and separated proteins were electroblotted onto nitrocellulose filters using Trans-Blot Turbo^TM^ Transfer System (Bio-Rad, Hemel Hempstead, United Kingdom). To verify loading and transfer efficiency, nitrocellulose filters were visualized with 0.2% (w/v) of Ponceau S stain (Sigma, p-3504) in 3% trichloroacetic solution for 1 min; filters showing dishomogeneity were discarded (Bragina et al., 2006). Nitrocellulose filters selected were finally probed with primary antibodies at dilutions as reported in **Table 1A**. After exposure to appropriate secondary antibodies (**Table 1B**), bands were visualized by Bio-Rad Chemidoc and Quantity One software using the SuperSignal West Pico (Rockford, IL, United States) chemiluminescent substrate (Bragina et al., 2006). Quantitation of immunoreactive bands were performed using *Analyze gels* function of ImageJ software (v. 1.48, NIH).

### Immunofluorescence

Levetiracetam-treated and control rats were anesthetized with chloral hydrate (300 mg/kg i.p.), and perfused transcardially with a flush of saline followed by freshly depolymerized 4% paraformaldehyde (PFA) in phosphate buffered saline (PB 0.1 M). Brains were removed, post-fixed in the same fixative for 24 h at 4°C, and cut with a vibratome into 50-μm-thick sections. Sections were incubated for 1 h in normal goat serum (NGS, 10% in PB with 0.3% Triton X-100) and then for 2 h at room temperature plus overnight at 4°C in a solution containing either VGLUT1, or VGLUT2 or VGAT primary antibodies (**Table 1A**). The next day, sections were incubated in NGS 10% (30 min) and in appropriate secondary fluorescent antibodies (**Table 1B**). Sections were then mounted, air-dried and coverslipped using Vectashield mounting medium (H-1000; Vector, Burlingame, CA, United States). For all experimental series (i.e., VGLUT1, VGLUT2, and VGAT), LEV-treated and control animals sections were run in parallel to minimize the variability of experimental conditions. Labeled sections were examined using a Leica TCS-SP2 confocal laser microscope equipped with an argon (488 nm) and a helium/neon (543 nm) laser. Images from all experimental series were from the parietal cortex, and were acquired from randomly selected subfields in layers II–VI (at least four fields for layer/animal). Supplemental fields from layer IV were acquired for VGLUT2 experimental series considering its particular layer distribution (Conti et al., 2005). Layer I was not sampled because it hardly contains VGAT+ puncta (Minelli et al., 2003). Sections from LEV-treated and control animals labeled for each antigen were acquired in parallel with the same confocal parameters, in order to minimize the variability of experimental conditions. Images were acquired using a 63× oil immersion lens (numerical aperture 1.4; pinhole 1.0 and image size 512 pixels × 512 pixels, yielding a pixel size of 0.155 μm) from a plane in which the resolution of both stains was optimal and never >1.8 μm from the surface (Melone et al., 2005). To improve the signal/noise ratio, 10 frames/image were averaged. Quantitative analysis was performed in ∼1000 randomly selected subfields measuring about 25 μm × 25 μm from the 512 pixels × 512 pixels images. Images were deconvolved using Iterative Deconvolve 3D plugin^1^ of ImageJ software (v. 1.48, NIH) with the same parameters for all images of both LEV-treated and control group; number and size of puncta were measured with the function *analyze particles* of the same software. Puncta size smaller than 4 pixels were excluded from the sample.

### Reverse Transcription-Polymerase Chain Reaction (RT-PCR)

Levetiracetam-treated and control rats were anesthetized with chloral hydrate (300 mg/kg i.p.) and decapitated, cerebral neocortex and hippocampus were quickly separated. Total RNA was extracted from whole hippocampus and cerebral neocortex after homogenization using TRIZOL reagent (Invitrogen, Milan, Italy), purified, digested with ribonuclease-free deoxyribonuclease and concentrated using RNeasy Micro kit (Qiagen, Milan, Italy) according to the respective manufacturer’s instructions. For determination of mRNA levels, 1 μg of RNA was reverse-transcribed with a High-Capacity cDNA RT Kit with RNase Inhibitor (Applied BioSystems, Foster City, CA, United States) in a total volume of 20 μl. Real time gene expression was analyzed in duplicate by using TaqMan Gene Expression Assays (**Table 1C**) and Master Mix TaqMan (Applied BioSystems, Foster City, CA, United States). Reactions were carried out in an ABI 7300 system (Applied BioSystems, Foster City, CA, United States) using 50 ng of RNA in a final reaction volume of 10 μl and the following thermal cycle protocol: initial incubation at 95°C 10 min, followed by 40 cycles of 95°C 15 s and 60°C 20 s. Technical duplicates were run for all samples and no RT and no template controls were included in all experiments. Stability comparisons of three candidate reference genes (*TBP*, β*-actin* and *HPRT-1*) were separately conducted for hippocampus and neocortex with the NormFinder algorithm^2^. The geometric mean of the most stable pair of genes was used as normalization factor for each sample. Relative mRNA expression was determined by the Δ*C*t method (2^-Δ^*^C^*^t^).

**Table 1C:** Taqman probes.

Target Gene	Source	Assay ID
ACTB	Applied BioSystems/Cat. #4453320	Rn00667869_m1
HPRT1	Applied BioSystems/Cat. #4453320	Rn01527840_m1
LRRK2	Applied BioSystems/Cat. #4448892	Rn01407714_m1
TBP	Applied BioSystems/Cat. #4453320	Rn01455646_m1
SYT1	Applied BioSystems/Cat. #4448892	Rn00436862_m1
SYT2	Applied BioSystems/Cat. #4448892	Rn00561994_m1
SYT9	Applied BioSystems/Cat. #4448892	Rn00584114_m1
SYN2	Applied BioSystems/Cat. #4448892	Rn00569739_m1
SYNGR1	Applied BioSystems/Cat. #4448892	Rn01505728_m1
SYNGR3	Applied BioSystems/Cat. #4448892	Rn01751300_m1
SLC17A6	Applied BioSystems/Cat. #4448892	Rn00584780_m1
SLC17A7	Applied BioSystems/Cat. #4448892	Rn01462431_m1
SLC32A1	Applied BioSystems/Cat. #4448892	Rn00824654_m1	
YWAHB	Applied BioSystems/Cat. #4448892	Rn00695953_m1	
YWAHE	Applied BioSystems/Cat. #4448892	Rn00494246_m1	

### Statistical Analysis

Statistical significance was evaluated by the non-parametric Mann–Whitney *U*-test using the GraphPad Prism Software (v. 6.0; GraphPad Software, San Diego, CA, United States).

### Network Analysis

We identified the interactions of the analyzed genes and proteins from eight databases: mentha; BioGrid; InnateDB; EBI-GOA-nonIntAct-MINT; Reactome-Fis; UniProt; BAR; InnateDB. Interactional data were merged and the interaction network was constructed using Cytoscape Software 3.4.0, redundant interactions were eliminated.

### Ethics Statement

All experimental procedures involving animals and their care were carried out in accordance with National laws and policies (D.L.26, March 14, 2014), and with the European Community Council Directive guidelines (2010/63/UE); all procedures were approved by the local authority veterinary services (Università Politecnica delle Marche).

## Results

**FIGURE 2:**
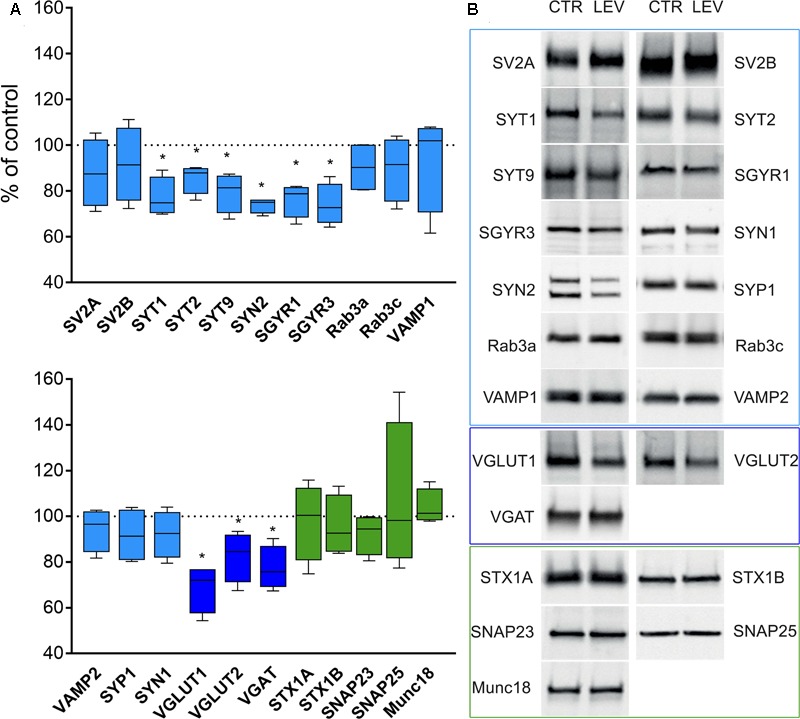
Levels of presynaptic proteins in crude synaptic membrane fraction (P3) of rat neocortex **(A,B)** in LEV treated animals (vesicular proteins, *light blue*; vesicular transporters, *blue*; and plasma membrane proteins, *green*). Values (mean ± SEM) are expressed as percentage of controls (dotted lines). ^∗^*P* < 0.05 (Mann–Whitney); *n* = 4 to 8 LEV, *n* = 4 to 8 CTR.

**FIGURE 3:**
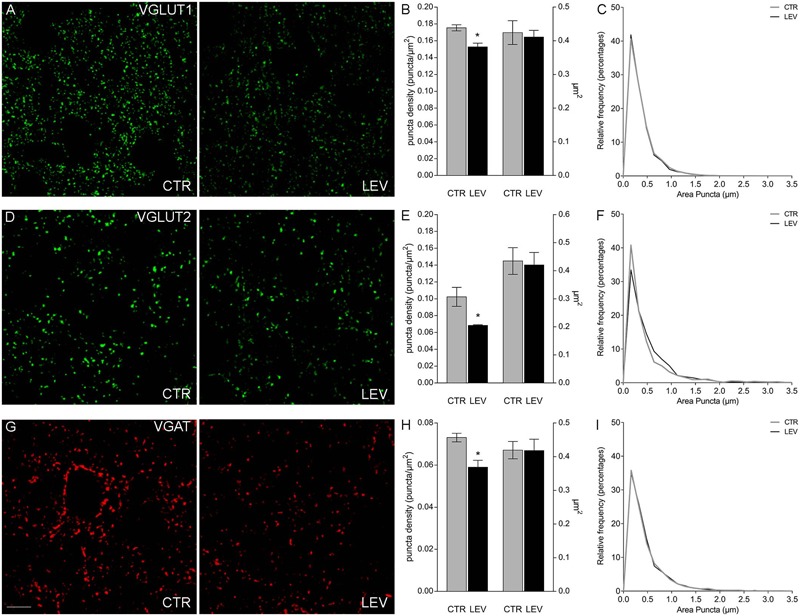
Confocal analysis of VGLUT1+ **(A)**, VGLUT2+ **(D)**, and VGAT+ **(G)** puncta in sections of rat cerebral cortex in LEV-treated (LEV; *n* = 4) and control animals (CTR; *n* = 4). Scale bar 10 μm. **(B,E,H)** Density (puncta/μm^2^; left axis) and mean size (μm^2^; right axis) of VGLUT1+ puncta of LEV (*black*) and control animals (gray). ^∗^*P* < 0.05 (Mann–Whitney). Density of VGLUT1, VGLUT2, and VGAT+ puncta were significantly reduced to 87.05% ± 2.58%, 66.83% ± 0.82%, and 80.78% ± 4.45% compared to controls in the order. ^∗^*P* < 0.05 (Mann–Whitney). **(C,F,I)** Since the average value can still hide differences in the distribution of positive puncta size (μm^2^), we compared the frequency distributions of VGLUT1+ **(C)**, VGLUT2+ **(F)**, and VGAT+ **(I)** puncta size of the control animal showing the highest puncta density (*black*) with the ones of the LEV-treated animal showing the lowest puncta density (gray), in order to maximize the possible effects produced by LEV.

We first measured the expression of vesicular proteins in neocortical crude membrane synaptic fractions (termed P3) (**Figure 1**) (Danbolt et al., 1990) of control and LEV-treated animals (**Figures 2A,B**, *light blue*). Quantitative analysis of independent samples showed that LEV treatment affected neither SV2A nor SV2B expression. Thus, LEV does not act by directly modifying the expression of its receptor; and its action does not induce secondary or compensatory changes in SV2A or SV2B levels. On the contrary, expression of SYTs was significantly reduced by LEV (*p* < 0.05); in particular, expression of SYT1, SYT2, and SYT9 was 77.10% ± 4.23%, 85.48% ± 3.22% and 79.43% ± 4.27% of controls, in the order. Significant changes following treatment were also observed for synapsin (SYN) II (73.79 ± 1.62%), SGYR1 (76.27% ± 3.68%), SGYR3 (73.92% ± 4.56%), VGLUT1 (68.81% ± 5.24%), VGLUT2 (82.60% ± 5.47%), and VGAT (77.33 ± 4.77%) (**Figures 2A,B**, *light blue* and *blue*). Levels of Rab3a, Rab3c, VAMP1, VAMP2, synaptophysin (SYP) I and SYNI were similar in both groups (**Figures 2A,B**, *light blue*), indicating that not all vesicular proteins considered here are altered by LEV. We also showed that in LEV-treated animals, expression of the major plasma membrane proteins participating in neurotransmitter release (i.e., STX1A, STX1B, SNAP23, SNAP25, and Munc18-1) were unchanged compared to controls (**Figures 2A,B**, *green*), an observation that highlights the central role of synaptic vesicles in LEV’s action. To verify whether LEV affected all terminals or a limited number of them, we studied the density (number of puncta/μm2) and the size (area in μm2) of VGLUT1, VGLUT2, and VGAT positive puncta, a simple and reliable method that is widely used to evaluate changes in the presynaptic compartment (Bozdagi et al., 2000; Antonova et al., 2001). The sections from LEV-treated and control animals were reacted in parallel with anti-VGLUT1, anti-VGLUT2 and anti-VGAT, and analyzed by confocal microscopy, as described earlier (Bragina et al., 2006) (**Figures 3A,D,G**). The results showed that their density was significantly reduced, whereas their size was unchanged compared to controls (**Figures 3B,C,E,F,H,I**). These studies showed that LEV does not act on all glutamatergic (either VGLUT1 or VGLUT2) or on all GABAergic terminals, in line with the observation that LEV exerts its effects only at active terminals (Meehan et al., 2011). WB analysis of the same vesicular and plasma membrane proteins was performed in hippocampal P3; results showed that LEV treatment did not affect their expression (data not shown).

**FIGURE 4:**
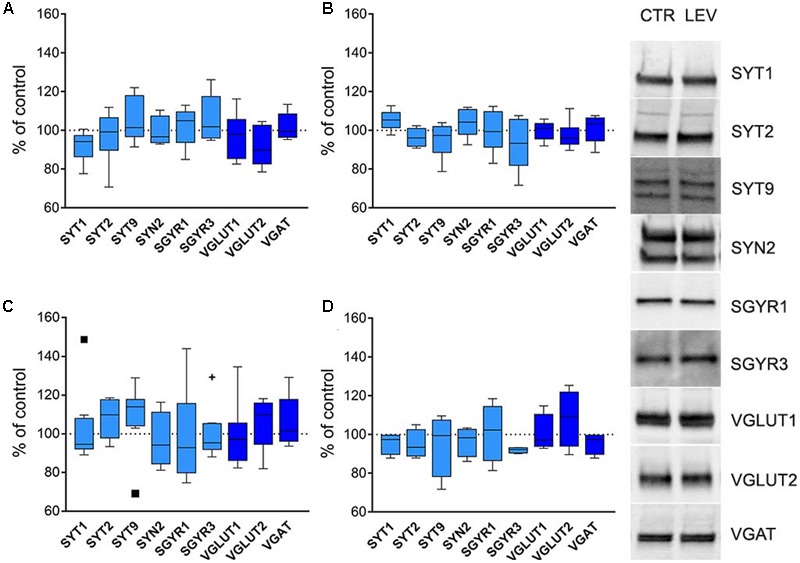
**(A,C)** Levels of mRNA coding for LEV-regulated proteins in rat neocortex **(A)** and hippocampus **(C)** of LEV treated animals (vesicular proteins, *light blue*; vesicular transporters, *blue*). Values (mean ± SEM) are expressed as percentage of controls (dotted lines). ^∗^*P* < 0.05 (Mann–Whitney); *n* = 8 LEV, *n* = 8 CTR. **(B,D)** Levels of LEV-regulated proteins in total proteins fraction (S1; excluding nuclei and debris) of rat neocortex **(B)** and hippocampus **(D)** of LEV-treated animals (vesicular proteins, *light blue*; vesicular transporters, *blue*). Values (mean ± SEM) are expressed as percentage of controls (dotted lines). Symbols in **(C)** identify outliers values. ^∗^*P* < 0.05 (Mann–Whitney); *n* = 4 to 8 LEV, *n* = 4 to 8 CTR.

Next, we asked whether LEV effects depended on transcriptional, translational or post-translational mechanisms. We therefore measured mRNA levels for LEV-regulated proteins, and analyzed WB of the same proteins in whole cellular proteins content devoid of nuclear fractions (termed S1) (Danbolt et al., 1990; Xu et al., 2013). In both neocortex and hippocampus of LEV-treated animals, mRNAs levels (**Figures 4A,C**) and S1 proteins expression (**Figures 4B,D**) were similar in the experimental and control groups, suggesting that LEV-induced changes are in all likelihood due to synaptic terminal-specific post-transcriptional mechanisms.

**FIGURE 5:**
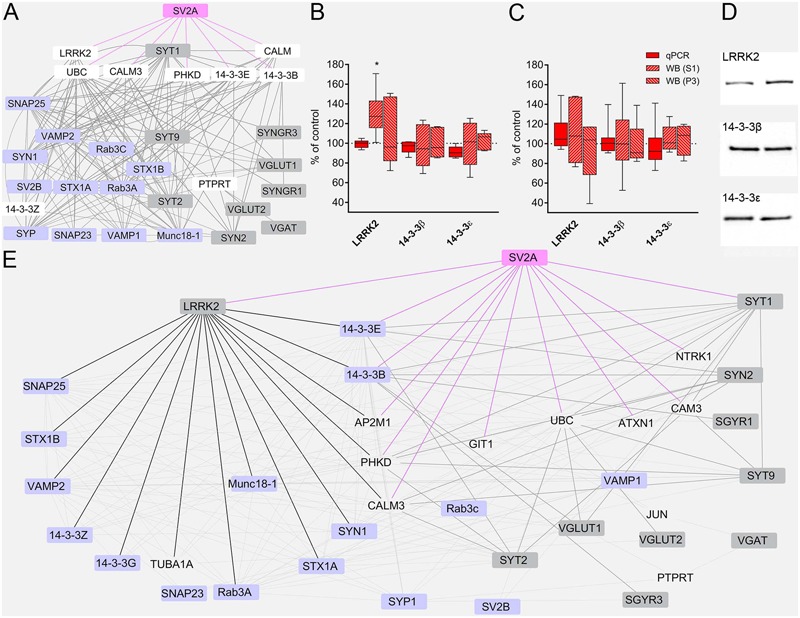
**(A)** The network of interactions of the presynaptic proteins investigated in the present study modeled from eight protein-protein interaction (PPI) databases. Nodes considered to be not relevant to our analysis or redundant are not shown. Regulated proteins, *gray nodes*; unregulated proteins, *violet nodes*; proteins not studied, *white nodes*; SV2A node and edges, *pink.* **(B,C)** RT-PCR of LRRK2 and 14-3-3s in rat neocortex **(B)** and hippocampus **(C)** of LEV treated animals. Levels of LRRK2 and 14-3-3s proteins in S1 samples of rat neocortex **(B,D)** and hippocampus **(C)** in LEV treated animals. Western blot showing levels of LRRK2 and 14-3-3s in P3 samples of rat neocortex **(B)** and hippocampus **(C)** in LEV treated animals. Values (mean ± SEM) are expressed as percentage of controls (dotted lines). ^∗^*P* < 0.05 (Mann–Whitney); *n* = 4 to 8 LEV, *n* = 4 to 8 CTR. **(E)** Network of analyzed PPI, querying for all proteins, including LRRK2, 14-3-3𝜀 and 14-3-3β. Nodes considered to be not relevant to our analysis or redundant are not shown. Regulated proteins, *gray nodes*; unregulated proteins, *violet nodes*; proteins not studied, *transparent nodes*; SV2A node and edges, *pink*; LRRK2 edges, *black*; modified proteins links to and from SV2A first degree interactors, *thick gray edges*.

To gain a deeper insight into LEV effects, we constructed a network of protein–protein interactions querying for the studied proteins. The analysis of the resulting network (**Figure 5A**) identified LRRK2 (leucine-rich repeat kinase 2, a large multidomain protein that includes a central catalytic tridomain with GTPase and kinase activities surrounded by a series of potential protein-protein interaction domains; Martin et al., 2014), 14-3-3β and 14-3-3𝜀 (14-3-3s are soluble proteins abundantly expressed in brain and involved in signal transduction, apoptotic, checkpoint control, and nutrient-sensing pathways by altering the subcellular localization of numerous binding partners; Aghazadeh and Papadopoulos, 2016) as SV2A interactors potentially capable of contributing to LEV effects. Therefore, we used RT-PCR and WB analysis to study mRNA and protein levels in neocortex. Results showed that mRNA levels coding for LRRK2 and 14-3-3s were not modified by LEV treatment (**Figure 5B**). WB studies showed that 14-3-3β and 𝜀 levels were not changed in S1 and P3 samples; and that LRRK2 protein levels were upregulated by LEV (up to 130.08 ± 9.35) in S1 but not in P3 samples, in line with its cellular localization (Martin et al., 2014) (**Figure 5B,D**). These findings indicate that LRRK2 up-regulation is mediated by a post-transcriptional mechanism. None of the mRNAs and proteins studied were modified by LEV in hippocampus, confirming the drug’s region-specific effect (**Figure 5C**). Finally, on a network re-analysis which included LRRK2 and 14-3-3s, we observed that LRRK2 never clustered with LEV-regulated proteins, while it strikingly clustered with most non-regulated presynaptic proteins, suggesting that LRRK2 plays a crucial role in defining the pattern of LEV effects (**Figure 5E**).

## Discussion

VGAT and the vast majority of VGLUT1 and VGLUT2 are expressed in axon terminals (e.g., Chaudhry et al., 1998; Kaneko et al., 2002; Minelli et al., 2003). However, VGLUT1 and VGLUT2 have also been described in some astrocytic processes (Bezzi et al., 2004; Montana et al., 2006; Ormel et al., 2012), and an astrocytic localization of most of the presynaptic proteins investigated here, including SV2, has also been described, albeit mostly *in vitro* (Montana et al., 2006), thus raising the possibility that a small part of the reported effects may be ascribed to astrocytes. In this context, it is worth noting that in Sanz-Blasco et al. (2016) reported that in astrocytic cultures LEV inhibits oligomeric Aβ-induced vesicular glutamate release. Accordingly, these data are compatible with the view that, perhaps, a minor part of the changes in presynaptic proteins induced by LEV may occur also in astrocytes.

All available evidence to date indicates that the synaptic vesicle protein SV2A is the only receptor for LEV (Lynch et al., 2004). On this basis, the results reported here indicate that LEV binding to SV2A down-regulates the expression of several SVs proteins in neocortex through a post-transcriptional mechanism, conceivably based on a protein-protein interaction network, in line with the reported LEV-induced reduction of release probability and quantal size (Wilson et al., 2005; Meehan et al., 2011; Li and Kavalali, 2017). The observations that SV2A is a regulator of SYT1 and SGYR1 levels (Yao et al., 2010); that modulation of presynaptic proteins results in reduced synaptic activity and release probability (Wilson et al., 2005; Meehan et al., 2011; Li and Kavalali, 2017); that deletion of SYNs, which are co-regulated with SV2A, abolishes LEV effectiveness (Boido et al., 2010); and that LEV is effective in epilepsy caused by Munc18-1 mutations (Dilena et al., 2016) are consistent with the present findings. In addition, we demonstrated that LEV affects neocortical but not hippocampal synapses. Considering that LEV action is activity-dependent, it is conceivable that its different effects in neocortex and hippocampus reflects their different pattern of activity (Ito et al., 2014), even though the different pattern of SV2A expression and function may also account for this selectivity (Venkatesan et al., 2012). Moreover, given that changes in the size of vesicular transporters positive puncta reflect the amount of protein expressed in terminals, while changes in density reflect the non-ubiquious action in all terninals, these studies are in line with the notion that LEV exerts its effects only at active terminals. Finally, we showed that LEV binding to SV2A up-regulates LRRK2, a proteostasis regulator (Martin et al., 2014), which is linked to most of the unregulated proteins of presynaptic interactome.

We used a dosing schedule that simulates chronic treatment in humans (Ueda et al., 2007). Considering the half-life of the studied proteins (Cohen et al., 2013), it is reasonable that the changes in protein levels require few days to take place. This could explain why LEV is not effective as a single-dose treatment in *status epilepticus* (Navarro et al., 2016).

The presynaptic protein-protein interaction network pointed out the centrality of 14-3-3β and 14-3-3𝜀 and LRRK2 in SV2A interactome. 14-3-3s are known to interact with multiple target proteins thereby interfering with protein folding and homeostasis (Aghazadeh and Papadopoulos, 2016). LRRK2 controls synaptic proteins levels by modulating autophagic proteins degradation, promoting translation (Martin et al., 2014), and accelerating endocytosis with regional and neuronal specificity (i.e., in GABAergic striatal neurons and not glutamatergic hippocampal neurons; Maas et al., 2017). Increased neocortical LRRK2, by regulating levels of LRRK2-linked proteins, may maintain normal concentrations of those proteins and, thus, may contribute to the pattern of LEV-induced regulations.

Levetiracetam-induced vesicular proteins down-regulation reported here may reduce synaptic strength of hyperactive terminals (Wilson et al., 2005; Xu et al., 2007; Meehan et al., 2011), thus preventing the probability of establishing epileptogenic circuits (Avramescu and Timofeev, 2008) and accounting for LEV neuroprotective effects (Löscher et al., 2016). Presynaptic proteins dysregulation is increasingly recognized in epilepsy (Li and Kavalali, 2017). Their differential involvement in the downstream mechanism of LEV effects may account for different responses to the drug administration in patients. In this context, it is worth noting that LEV reduces seizures in Munc18-1-related epileptic encephalopathy, which is refractory to other antiepileptic drugs (Dilena et al., 2016). According to our findings, this effect may depend on the fact that Munc18-1 is not among the proteins modulated by the interaction between LEV and SV2A. Therefore, LEV may conceivably be a therapeutic tool for those patients with mutations of presynaptic proteins not regulated by LEV that determine epileptic syndromes.

Furthermore, reduction of synaptic strength by SVs proteins down-regulation induced by LEV may also protect against abnormal and hypersynchronous brain activity (Bakker et al., 2012; Sanchez et al., 2012; Devi and Ohno, 2013; Shi et al., 2013; Nygaard et al., 2015), an early marker and a progression factor of Alzheimer’s disease (Bezzina et al., 2015). Moreover, Baker et al. (2015) describe a human condition with dyskinetic movement disorder, severe motor delay and profound cognitive impairment associated with a rare variant in SYT1. In addition, in a senescence-accelerated prone mouse 8 (SAMP8), Chen et al. (2007) demonstrated a positive correlation between SYT1 level and age-related cognitive impairment. Finally, Ohrfelt et al. (2016) demonstrate that, in cerebrospinal fluid, SYT1 levels are significantly higher in patients with dementia or mild cognitive impairment due to Alzheimer’s disease compared to controls. On these bases, it is conceivable to extend LEV treatment also in age-related cognitive impairment and in Alzheimer’s disease.

## Conclusion

The presynaptic proteins regulation induced by LEV reported here claims that not only SV2A, but the interactions between presynaptic proteins downstream of SV2A, actually mediate LEV effects; and that LRRK2 plays a role in forging the underlying pattern of molecular changes.

## Author Contributions

GF and DM conceived the project. GF, DM, JP, and LB performed the experiments, and gathered and analyzed the data. FC supervised the project, and discussed the data. GF, DM, and FC wrote the paper.

## Conflict of Interest Statement

The authors declare that the research was conducted in the absence of any commercial or financial relationships that could be construed as a potential conflict of interest.

## Footnotes

1 http://www.optinav.info/Iterative-Deconvolve-3D.htm

2 https://moma.dk/normfinder-software
